# Predictors of Short-Term Outcome in Venous Leg Ulcers: The Dominant Role of Wound Infection and Multiple Ulcers

**DOI:** 10.3390/jcm15197525

**Published:** 2026-09-27

**Authors:** Daciana Elena Branisteanu, Oana Mihaela Condurache Hritcu, Catalina Onu-Branisteanu, Daniel Constantin Branisteanu, Stefan Toader, Roxana-Paraschiva Ciobanu, Elena Porumb-Andrese, Mihaela Paula Toader

**Affiliations:** 1 Department of Dermatology, Faculty of Medicine, Grigore T. Popa University of Medicine and Pharmacy, 700115 Iasi, Romania; debranisteanu@yahoo.com (D.E.B.); elena.andrese1@umfiasi.ro (E.P.-A.); 2 Dermatology Clinic, University Clinical Railways Hospital, 700506 Iasi, Romania; r.p.ciobanu@gmail.com (R.-P.C.); toaderpaula@gmail.com (M.P.T.); 3 Department of Surgicals, Faculty of Dental Medicine, Grigore T. Popa University of Medicine and Pharmacy, 700115 Iasi, Romania; toaderstefan@gmail.com; 4 Institute for Cardiovascular Diseases C.C. Iliescu, 258 Fundeni St., 022328 Bucharest, Romania; branisteanucatalina@yahoo.com; 5 Department of Surgery II-Ophthalmology, Grigore T. Popa University of Medicine and Pharmacy, 700115 Iasi, Romania; daniel.branisteanu@umfiasi.ro; 6 Ophtalmology Clinic, University Clinical Railways Hospital, 700506 Iasi, Romania

**Keywords:** venous leg ulcer, CEAP C6, wound infection, multiple ulcers, prognosis, neutrophil-to-lymphocyte ratio

## Abstract

**Background/Objectives:** CEAP C6 venous leg ulcers are the most resource-intensive stage of chronic venous disease, yet short-term prognostication at admission remains empirical. We aimed to determine which routinely available admission variables are associated with unfavourable in-episode evolution and to develop a concise prognostic model. **Methods:** Single-centre retrospective observational cohort of 87 consecutive patients; in-episode evolution (favourable versus unfavourable) was documented in 59. Associations were tested by univariate methods and binary logistic regression. Because outcome was undocumented in 32.2% of the cohort, a tipping-point sensitivity analysis quantified the departure from a missing-at-random mechanism required to nullify each association. **Results:** Wound infection was recorded in 69.0% of patients, with *Pseudomonas* spp. and meticillin-sensitive *Staphylococcus aureus* predominating. Evolution was unfavourable in 34/59 patients (57.6%) and was associated with wound infection (OR 7.00, 95% CI 2.13–23.03), microbial eczema, multiple ulceration and urban residence, but with neither the neutrophil-to-lymphocyte ratio (*p* = 0.920) nor any individual comorbidity. In the final model, wound infection (OR 6.10), urban residence (OR 5.81) and multiple ulceration (OR 5.01) were independently associated with outcome (Nagelkerke R^2^ = 0.405; AUC 0.812, 95% CI 0.704–0.919). Sensitivity analysis showed the wound infection association to be robust to informative missingness, whereas urban residence was the most fragile. **Conclusions:** Wound-level factors—infection and multiple ulcers—carried more short-term prognostic information than comorbidity burden or systemic inflammatory indices. These findings are hypothesis-generating and require external validation.

## 1. Introduction

Chronic venous disease culminates, in its most advanced clinical class, in venous leg ulceration (Clinical–Etiological–Anatomical–Pathophysiological [CEAP] class C6) [1,2]. Venous leg ulcers are common, with pooled prevalence around 1% of adults and a steep rise with age [3], and they represent the most severe and resource-intensive stage of the disease [4,5]. Their natural history is dominated by chronicity and relapse: many fail to heal within months and a large fraction recur [6,7], with recurrence closely tied to self-efficacy and psychosocial support [8]. The resulting burden—pain, exudate, malodour, impaired mobility and reduced quality of life—falls disproportionately on older, comorbid patients [5,9].

The dominant pathophysiological model begins with ambulatory venous hypertension arising from valvular incompetence or calf-pump failure, which at the microvascular level distends capillaries, disrupts the endothelial glycocalyx and increases permeability [10,11,12]. Sustained inflammation, leukocyte trafficking, cytokine and matrix-metalloproteinase (MMP) release, reactive oxygen species and iron deposition lock the wound bed into a chronic, proteolytic state that degrades the extracellular matrix and stalls keratinocyte migration and epithelialisation [13,14,15,16]; these healing processes are governed by proteases, cytokines such as interleukin-6, and fibroblast and keratinocyte function [17,18]. Accurate diagnosis rests on clinical and haemodynamic assessment, with exclusion of non-venous contributors [19,20].

Against this background, two distinct “burdens” are commonly considered to contribute to the refractory course of some C6 ulcers. The first is the wound’s microbiology. Most C6 ulcers are colonised; the clinically important transition is from colonisation to wound infection, accompanied by biofilm formation, bacterial persistence, and a self-perpetuating inflammatory response that further delays epithelialisation [21,22,23,24]. *Pseudomonas aeruginosa* and *Staphylococcus aureus* dominate the isolate spectrum of C6 ulcers, frequently alongside other Gram-negative bacilli, and antimicrobial resistance is an increasing concern [25,26,27,28]. *P. aeruginosa*-colonised wounds tend to be larger and to heal poorly [21], bacterial bioburden has been linked to delayed healing [29], and the local microenvironment, including wound pH, shapes the bacterial profile [30].

The second is host multimorbidity. C6 patients are typically older and carry a heavy load of cardiometabolic disease—hypertension, heart failure, diabetes and obesity—alongside reduced mobility, lymphoedema and nutritional compromise [31,32,33,34,35]. Systemic inflammation has accordingly been proposed as a unifying prognostic signal: circulating cytokine and chemokine networks are demonstrably altered in chronic venous disease [36,37], and candidate wound-fluid and blood biomarkers have been linked to healing [38,39,40,41]. The neutrophil-to-lymphocyte ratio (NLR) is an inexpensive, universally available index of systemic inflammation, but evidence linking such surrogates, or a simple comorbidity count, specifically to the course of an established ulcer rather than to its development or recurrence remains inconsistent [42].

These two accounts have rarely been evaluated together, within the same cohort, against a clinically defined short-term outcome. Most studies report either microbiological or host-level correlates, and many report severity scores rather than evolution. Clinicians caring for hospitalised C6 patients, however, need to know which routinely available variables at admission identify those unlikely to improve during the current episode.

We therefore studied a consecutive single-centre cohort of patients admitted with CEAP C6 ulceration and examined which demographic, ulcer-related, microbiological, biological, therapeutic and historical variables are associated with unfavourable in-episode evolution, and whether a concise multivariable model built from routinely collected data can discriminate between favourable and unfavourable courses.

## 2. Materials and Methods

### 2.1. Design, Setting and Population

This retrospective single-centre observational cohort study [43] was conducted at the Railways University Clinical Hospital, Iași, Romania, between January 2021 and December 2025. Consecutive patients were eligible if they were aged ≥ 18 years and had an active lower-limb venous ulcer classified as CEAP C6 at admission. Venous aetiology and clinical classification were established by combined clinical and haemodynamic assessment. Patients were excluded if the ulcer was predominantly arterial, neuropathic, pressure-related, traumatic, vasculitic, neoplastic or otherwise of non-venous origin; if haemodynamic findings did not support a venous aetiology; or if the available records were insufficient to confirm CEAP C6 status or extract the predefined study variables. In patients with repeated admissions, only the first episode was used for the primary patient-level analysis, while the total number of admissions was retained as a separate historical variable. Eighty-seven consecutive eligible patients were included (Figure 1).

### 2.2. Unit of Analysis

The patient was the unit of analysis throughout (*n* = 87). Where a patient had been admitted more than once, clinical, microbiological and biological variables refer to the first episode, and the total number of admissions was recorded as a separate patient-level variable. No presentation-level or repeated-measures analysis was undertaken.

### 2.3. Variables and Definitions

Age was recorded as decade of life (fourth to ninth) and treated as an ordinal variable. Residence was classified as urban or rural. Ulcer morphology was recorded as single or multiple, as circumferential or non-circumferential, and by maximum diameter (<2 cm, 2–6 cm, >6 cm). Venous severity was scored using the revised Venous Clinical Severity Score (VCSS) [44] and pain using a 0–10 visual analogue scale (VAS).

Microbial eczema and cellulitis were diagnosed clinically. Wound infection was diagnosed clinically based on local signs, including hypergranulation, friable or bleeding granulation tissue, increased exudate, perilesional erythema, edema and warmth, wound breakdown or enlargement, purulent discharge, increased pain, and malodour. Signs of spreading infection, including progressive induration, erythema extending >2 cm beyond the wound margin, and lymphangitis, were also assessed. Wound culture with antibiogram was considered supportive microbiological information and was not, in isolation, sufficient to establish the diagnosis of wound infection. Accordingly, culture positivity was not considered synonymous with clinical wound infection, as bacterial colonisation is common in chronic venous ulcers. Because of the retrospective design, a prospectively standardised set of diagnostic criteria for wound infection was not available. Antibiogram phenotype was dichotomised as multi-sensitive or multidrug-resistant (MDR). Extra-ulcer infectious foci comprised nasal and pharyngeal carriage screening and any other clinically documented focus, most commonly dermatophyte infection.

Acute-phase reactants (erythrocyte sedimentation rate [ESR], CRP, fibrinogen), the lipid panel and fasting glucose were available only as qualitative categories (elevated, normal, low) and are summarised as proportions; the NLR was recorded as a continuous variable. Comorbidities were considered present if documented at any point, and were additionally coded according to whether they antedated the onset of chronic venous disease.

The outcome was in-episode evolution, recorded by the treating clinician in four categories—healed, improved, stationary, worsened—and dichotomised a priori as favourable (healed or improved) versus unfavourable (stationary or worsened). Outcome was documented as part of routine care and was not assessed blind to the patient’s clinical, microbiological or laboratory status; candidate predictors were likewise recorded without blinding to outcome.

### 2.4. Missing Data

No imputation was performed and denominators are reported throughout. Evolution was documented in 59/87 patients (67.8%); the remaining 28 patients (32.2%) had no recorded outcome and were excluded from all prognostic analyses. Other variables with substantial missingness included duration of chronic venous disease (*n* = 33), time from disease onset to C6 (*n* = 31), current ulcer duration (*n* = 62), VCSS (*n* = 65), VAS pain (*n* = 72) and NLR (*n* = 84).

### 2.5. Statistical Analysis

Categorical variables are presented as counts and percentages, and continuous variables as mean ± standard deviation together with median, interquartile range (IQR) and range. Comparisons between the favourable and unfavourable groups used the Pearson chi-squared test for categorical variables, with Fisher’s exact test substituted where expected cell counts were below five, and the Mann–Whitney U test for continuous variables, which were not assumed to be normally distributed. Unadjusted odds ratios with 95% confidence intervals were calculated for dichotomous variables reaching statistical significance.

Candidate predictors for multivariable modelling were restricted to variables that were significant on univariate analysis, were available at or near admission, and were not treatment variables, since the latter are subject to confounding by indication and cannot be interpreted prognostically in an observational design. Two univariately significant variables—elevated CRP and the presence of extra-ulcer infectious foci—were additionally excluded because of substantial missingness, which would have destabilised the model.

With 34 unfavourable and 25 favourable outcomes, an events-per-variable constraint of at least ten permitted a maximum of three to four predictors. Collinearity among the remaining candidates was assessed by cross-tabulation and Cramér’s V; where a pair exceeded V = 0.5, only one member was retained, selected on the basis of a larger effect estimate, a narrower confidence interval and greater clinical breadth.

A binary logistic regression model was then fitted with the enter method. Model adequacy was assessed by the Omnibus test of model coefficients, calibration by the Hosmer–Lemeshow goodness-of-fit test, explained variance by Nagelkerke R^2^, and classification performance by the classification table at the default 0.5 cut-off compared with the null model. A non-significant predictor was removed and the model refitted to improve parsimony and the events-per-variable ratio. Discrimination of the final model was quantified by ROC analysis of predicted probabilities, reporting the area under the curve (AUC) with its 95% CI (non-parametric method), and the optimal cut-off by the Youden index with corresponding sensitivity and specificity.

Exploratory analyses stratified by ulcer morphology (single versus multiple; circumferential versus non-circumferential) and by ulcer size were performed using the same univariate methods. Given stratum sizes of 7 to 52 patients and the absence of adjustment for multiplicity, these were regarded as hypothesis-generating and are reported in summary only.

Because in-episode evolution was undocumented in 32.2% of the cohort, we did not rely on a missing-at-random assumption alone. For each variable retained in the final model we performed a tipping-point sensitivity analysis: the 28 patients without a recorded outcome were assigned across the full range of possible unfavourable-outcome rates, separately for those with and without the factor concerned, and the association was recomputed at every point on this grid. We report the boundary at which statistical significance is lost and the proportion of the resulting scenario space in which a significant positive association is retained. Because the exposure status of the undocumented patients was known for all three model variables, these bounds are exact rather than assumed.

Since model coefficients estimated in small samples are optimistically biased, we additionally computed the van Houwelingen–Le Cessie heuristic shrinkage factor, (model χ^2^ − df)/model χ^2^, and report shrunken odds ratios alongside the maximum-likelihood estimates. The precision attainable with the available sample was characterised by the minimum odds ratio detectable at 80% power for a two-group comparison of the observed size.

All tests were two-sided with α = 0.05. Values with 0.05 ≤ *p* < 0.10 are reported as trends and are not interpreted as evidence of association. Analyses were performed in IBM SPSS Statistics version 31.0 (IBM Corp., Armonk, NY, USA).

## 3. Results

### 3.1. Cohort Characteristics

The cohort comprised 87 patients, of whom 50 (57.5%) were men and 46 (52.9%) were urban residents. Age was concentrated in the sixth to eighth decades, which together accounted for 68/87 patients (78.2%). For 31 patients (35.6%) the study centre was the first point of medical contact for the ulcer, and 78 patients (89.7%) were managed as continuous inpatient admissions. Mean length of stay was 6.08 ± 1.76 days and the mean number of admissions was 2.87 ± 2.66 (median 2, IQR 1–4, range 1–14). Patient and ulcer characteristics are shown in Table 1.

Ulceration was single in 52 patients (59.8%) and multiple in 35 (40.2%); 10 ulcers (11.5%) were circumferential. Maximum ulcer diameter was below 2 cm in 42 patients (48.3%), 2–6 cm in 24 (27.6%) and above 6 cm in 21 (24.1%). Median VCSS was 11.0 (IQR 10.0–16.0; mean 13.20 ± 4.48; range 6–26; *n* = 65) and median VAS pain 5.0 (IQR 4.0–7.0; mean 5.40 ± 1.93; *n* = 72). Median NLR was 2.93 (IQR 2.24–3.95; mean 3.30 ± 1.60; range 0.92–9.34; *n* = 84) (Table 2).

### 3.2. Complications, Microbiology and Infectious Foci

Eczema was present in 71 patients (81.6%), microbial eczema in 50 (57.5%), wound infection in 60 (69.0%) and cellulitis in 8 (9.2%). Thromboembolic complications during the episode were rare: superficial thrombophlebitis in 2 patients (2.3%), deep thrombophlebitis in 1 (1.1%) and pulmonary embolism in 1 (1.1%). Four patients (4.6%) were referred to infectious diseases.

Among the 60 patients with wound infection, *Pseudomonas* spp. was the most frequently identified organism (11 patients, 18.3%), followed by methicillin-sensitive *S. aureus* (MSSA; 8 patients, 13.3%), MSSA together with *Pseudomonas* (5, 8.3%), methicillin-resistant *S. aureus* (MRSA; 4, 6.7%), and *Acinetobacter* spp., *Escherichia coli*, *S. aureus* (unspecified sensitivity) and coagulase-variable staphylococci (3 each, 5.0%). Antibiogram phenotype was multi-sensitive in 48 patients (80.0%), multidrug-resistant in 8 (13.3%) and not available in 4 (6.7%). The isolate distribution in microbial eczema was closely similar, with *Pseudomonas* spp. in 10/50 (20.0%) and MSSA in 6/50 (12.0%); 8/50 isolates (16.0%) were multidrug-resistant. Among the 8 patients with cellulitis, *Pseudomonas* spp. was identified in 3 (37.5%) and the multidrug-resistant proportion was higher (3/8, 37.5%), although this subgroup is too small to support inference (Table 3).

Nasal carriage screening was positive in 12/62 tested patients (19.4%), most often MSSA (8/12, 66.7%) with MRSA in 2 (16.7%); all nasal isolates were multi-sensitive. Pharyngeal screening was negative in all 62 patients tested. Other infectious foci were documented in 33 patients (37.9%), predominantly onychomycosis (13, 39.4%) and tinea pedis (13, 39.4%, with a further 3 cases of tinea pedis with intertrigo, 9.1%).

### 3.3. Comorbidity, Biological and Treatment Profile

Hypertension was the commonest comorbidity (69 patients, 79.3%), followed by obesity (39, 44.8%), congestive heart failure (28, 32.2%), diabetes or impaired fasting glucose (21, 24.1%), depression (16, 18.4%), overweight (13, 14.9%), anxiety (11, 12.6%), compulsive eating (10, 11.5%), alcohol use (9, 10.3%) and smoking (7, 8.0%). Hypertension antedated the onset of chronic venous disease in 21 patients (24.1%) and obesity in 12 (13.8%).

ESR was qualitatively elevated in 53 patients (60.9%), CRP in 35 (40.2%), fibrinogen in 14 (16.1%) and fasting glucose in 11 (12.6%). Lipid abnormalities were infrequent: elevated LDL cholesterol in 13 patients (14.9%), elevated total cholesterol in 12 (13.8%), elevated triglycerides in 10 (11.5%) and low HDL cholesterol in 9 (10.3%).

Systemic antibiotics were administered to 41 patients (47.1%), most commonly moxifloxacin (15/41, 36.6%) and ciprofloxacin (6/41, 14.6%), for a mean of 7.88 ± 1.68 days. Topical antibiotics were used in 7 patients (8.0%). Antiseptic irrigation with polyhexanide or hypochlorous acid solution was near-universal (83 patients, 95.4%) and iodoform was used in 18 (20.7%). Enzymatic debridement was performed in 47 patients (54.0%) over a mean of 6.78 ± 2.90 days, and surgical debridement in 2 (2.3%). Re-epithelialising agents were used in 49 patients (56.3%), topical corticosteroids in 44 (50.6%), fixed corticosteroid–antibiotic combinations in 17 (19.5%), polarised light in 23 (26.4%) and advanced dressings in 11 (12.6%). Adjuvant measures included postural drainage in all 87 patients (100%), hydrotherapy in 70 (80.5%), antihistamines in 53 (60.9%) and instrumental lymphatic drainage in 20 (23.0%).

### 3.4. Outcome

In-episode evolution was documented in 59/87 patients (67.8%): 8 patients healed (9.2% of the whole cohort), 17 improved (19.5%), 27 remained stationary (31.0%) and 7 worsened (8.0%). Dichotomised, evolution was favourable in 25 patients and unfavourable in 34, so that 34/59 (57.6%) of patients with recorded outcome had an unfavourable course. Among the 8 patients who healed, mean time to healing was 5.25 ± 3.41 months (median 4.50, IQR 2.25–7.50, range 2–12). Where worsening was documented (*n* = 7), the recorded explanation was an increase in the number of ulcers in 3 patients (42.9%), an increase in ulcer size in 2 (28.6%) and infrequent attendance in 2 (28.6%).

### 3.5. Univariate Analysis of Factors Associated with Unfavourable Evolution

Univariate results are presented in Table 4. Unfavourable evolution was significantly associated with urban residence (23/34, 67.6% versus 10/25, 40.0%; χ^2^ = 4.468, *p* = 0.035; OR 3.14, 95% CI 1.07–9.19), multiple ulceration (17/34, 50.0% versus 5/25, 20.0%; χ^2^ = 5.545, *p* = 0.019; OR 4.00, 95% CI 1.22–13.16), microbial eczema (24/34, 70.6% versus 8/25, 32.0%; χ^2^ = 8.643, *p* = 0.003; OR 5.10, 95% CI 1.67–15.60), wound infection (28/34, 82.4% versus 10/25, 40.0%; χ^2^ = 11.273, *p* < 0.001; OR 7.00, 95% CI 2.13–23.03), elevated CRP (15/29, 51.7% versus 3/20, 15.0%; χ^2^ = 6.869, *p* = 0.009; OR 6.07, 95% CI 1.46–25.30) and the presence of extra-ulcer infectious foci (16/23, 69.6% versus 5/20, 25.0%; χ^2^ = 8.503, *p* = 0.004; OR 6.86, 95% CI 1.78–26.36). The number of admissions was higher in the unfavourable group (4.50 ± 3.36 versus 2.56 ± 1.47; U = 258.0, *p* = 0.009).

Three variables showed trends that did not reach significance: VCSS (14.10 ± 5.27, *n* = 29, versus 11.07 ± 2.96, *n* = 15; U = 149.5, *p* = 0.090), a positive nasal swab (8/21, 38.1% versus 1/15, 6.7%; χ^2^ = 4.610, *p* = 0.051) and elevated fibrinogen (8/19, 42.1% versus 2/16, 12.5%; χ^2^ = 3.730, *p* = 0.071).

No association was found with sex (*p* = 0.589), decade of life (*p* = 0.399), admission type (*p* = 0.687), circumferential morphology (*p* = 0.687), ulcer size category (*p* = 0.278), length of stay (*p* = 0.272), duration of chronic venous disease (*p* = 0.764), time to C6 (*p* = 0.645), duration of the current ulcer (*p* = 0.112), VAS pain (*p* = 0.977) or NLR (3.36 ± 1.82 versus 3.24 ± 1.40; U = 373.5, *p* = 0.920). None of the ten recorded comorbidities was associated with outcome, whether analysed as ever-present or as antedating chronic venous disease (all *p* ≥ 0.109). BMI was routinely recorded for all patients, with overweight and obesity additionally classified as categorical comorbidities. Neither obesity nor overweight was significantly associated with short-term outcome (*p* = 0.788 and *p* = 0.445, respectively). In additional analyses examining their relationship with residence, neither obesity (*p* = 0.069; Fisher’s exact *p* = 0.100) nor overweight (*p* = 0.787) was significantly associated with urban residence. ESR (*p* = 0.344), fasting glucose (*p* = 0.389) and all lipid fractions (*p* ≥ 0.114) were likewise unassociated, as were all variables describing previous thromboembolic events, previous ulceration or treatment, varicose vein status and prior vascular surgical referral (all *p* ≥ 0.207).

Five treatment variables were significantly associated with outcome: systemic antibiotic administration (OR 10.98, 95% CI 3.03–39.81; *p* < 0.001), iodoform use (OR 11.48, 95% CI 1.37–96.16; *p* = 0.009), polarised light (OR 4.54, 95% CI 1.13–18.24; *p* = 0.025), antihistamines (OR 3.60, 95% CI 1.21–10.69; *p* = 0.019) and instrumental lymphatic drainage, which was associated with a lower odds of unfavourable evolution (OR 0.17, 95% CI 0.05–0.63; *p* = 0.005). These variables were not eligible for prognostic modelling (Section 2.5) and are reported for completeness only.

### 3.6. Collinearity Screening and Multivariable Model

Of the univariately significant variables, elevated CRP and extra-ulcer infectious foci were excluded because of missingness, and treatment variables were excluded by design, leaving urban residence, multiple ulceration, number of admissions, microbial eczema and wound infection as candidates. Pairwise Cramér’s V values were 0.118 for residence and ulcer number, 0.119 for residence and microbial eczema, 0.014 for residence and wound infection, 0.184 for ulcer number and microbial eczema, 0.196 for ulcer number and wound infection, and 0.730 for microbial eczema and wound infection. The last pair exceeded the pre-specified threshold of 0.5; wound infection was retained, having the larger unadjusted effect estimate, the narrower confidence interval and the broader clinical definition.

The initial four-predictor model was adequate (Omnibus χ^2^ = 23.278, df = 4, *p* < 0.001; Hosmer–Lemeshow χ^2^ = 5.825, df = 5, *p* = 0.560; Nagelkerke R^2^ = 0.438; overall accuracy 72.9%, 68.0% for favourable and 76.5% for unfavourable evolution). Urban residence (OR 4.76, 95% CI 1.14–19.88; *p* = 0.032), multiple ulceration (OR 5.09, 95% CI 1.12–23.05; *p* = 0.035) and wound infection (OR 4.26, 95% CI 1.05–17.27; *p* = 0.042) were significant, whereas the number of admissions was not (OR 1.23, 95% CI 0.90–1.69; *p* = 0.196).

The number of admissions was therefore removed and the model refitted with three predictors. The reduced model remained adequate and better satisfied the events-per-variable constraint (Omnibus χ^2^ = 21.176, df = 3, *p* < 0.001; Hosmer–Lemeshow χ^2^ = 4.077, df = 5, *p* = 0.538; Nagelkerke R^2^ = 0.405; overall accuracy 72.9%, 72.0% for favourable and 73.5% for unfavourable evolution, against 57.6% for the null model). All three predictors were significant: urban residence OR 5.81 (95% CI 1.42–23.75; *p* = 0.014), multiple ulceration OR 5.01 (95% CI 1.12–22.46; *p* = 0.035) and wound infection OR 6.10 (95% CI 1.63–22.88; *p* = 0.007) (Table 5, Figure 2).

The three regression coefficients were closely similar in magnitude (β = 1.761, 1.611 and 1.808 for urban residence, multiple ulceration and wound infection respectively), corresponding to a mean odds ratio of 5.62 per factor present. The van Houwelingen–Le Cessie heuristic shrinkage factor was (21.176 − 3)/21.176 = 0.858, giving shrunken odds ratios of 4.53 for urban residence, 3.99 for multiple ulceration and 4.72 for wound infection.

### 3.7. Model Discrimination

ROC analysis of the predicted probabilities generated by the final three-variable logistic regression model demonstrated good discriminative performance, with an area under the curve (AUC) of 0.812 (95% CI 0.704–0.919; *p* < 0.001), indicating good discrimination between favourable and unfavourable in-episode outcomes.

Given the lower robustness of the urban-residence association in the sensitivity analysis, an exploratory reduced model including only wound infection and multiple ulceration was also fitted. The reduced model remained statistically significant (Omnibus χ^2^ = 14.18, df = 2, *p* < 0.001; Nagelkerke R^2^ = 0.287). Wound infection remained independently associated with unfavourable evolution (OR 5.73, 95% CI 1.68–19.50; *p* = 0.005), whereas multiple ulceration did not reach statistical significance (OR 2.86, 95% CI 0.80–10.28; *p* = 0.108). The reduced model had an AUC of 0.761 (95% CI 0.641–0.882), compared with 0.812 (95% CI 0.704–0.919) for the three-predictor model.

### 3.8. Exploratory Stratified Analyses

Analyses stratified by ulcer morphology and size reproduced the direction of the principal associations, with confidence intervals too wide for the magnitudes to be estimated usefully. In the single-ulceration (*n* = 37) and non-circumferential (*n* = 52) strata, microbial eczema, wound infection and elevated CRP each remained significantly associated with unfavourable evolution. In the multiple-ulceration stratum (*n* = 22) only systemic antibiotic use reached significance, and the circumferential (*n* = 7) and size-based strata (*n* = 16–26) were too small to interpret. Full stratified results are available from the corresponding author on request and are not interpreted further.

### 3.9. Sensitivity of the Findings to Undocumented Outcomes

Outcome was undocumented in 28 patients, of whom 22 had wound infection, 13 urban residence and 13 multiple ulceration. Under a missing-at-random assumption—that is, assigning the undocumented patients the outcome rates observed within their exposure group—all three associations strengthened (wound infection *p* < 0.001, multiple ulceration *p* = 0.004, urban residence *p* = 0.010), reflecting the larger effective sample.

Tipping-point analysis showed markedly different robustness across the three variables (Table 6, Figure 3). The association with wound infection was the most resistant to informative missingness: the unfavourable rate among the 22 undocumented patients with wound infection would have to fall from the observed 73.7% to 11.7% before significance was lost, and the association persisted even if every undocumented patient without wound infection had experienced an unfavourable course. A significant positive association was retained across 74.4% of the scenario space. Multiple ulceration was intermediate, with tipping points at 50.3% (from 77.3%) and 83.9% (from 45.9%) and 47.4% of the scenario space retained. Urban residence was the most fragile: the association was nullified once the unfavourable rate among undocumented urban patients fell to 46.7%, a departure of only 23 percentage points from the observed 69.7%, with 45.5% of the scenario space retained.

Each column reports the unfavourable-outcome rate that would have to obtain among the undocumented patients, holding the other group at its observed rate, before the univariate association ceases to reach *p* < 0.05. “Scenario space retained” is the proportion of all combinations of the two rates across the unit square in which a significant positive association persists.

## 4. Discussion

In this consecutive single-centre cohort of 87 patients admitted with CEAP C6 venous ulceration, unfavourable short-term evolution was common, occurring in 34 of the 59 patients (57.6%) in whom outcome was documented. Three variables available at admission were independently associated with that outcome: wound infection, multiple ulceration and urban residence. The resulting three-variable model explained approximately 40% of outcome variance and discriminated acceptably between courses (AUC 0.812). An exploratory model restricted to wound infection and multiple ulceration showed lower discrimination than the three-predictor model (AUC 0.761 versus 0.812), suggesting that urban residence contributed incremental prognostic information despite its greater sensitivity to assumptions regarding undocumented outcomes. Equally informative are the negative findings. Neither the NLR nor any individual comorbidity—including hypertension, heart failure, diabetes and obesity—showed any association with outcome, and venous severity as measured by VCSS showed only a non-significant trend.

### 4.1. Wound Infection as the Dominant Modifiable Correlate

That wound infection, rather than host comorbidity or systemic inflammatory indices, emerged as the strongest correlate of an unfavourable course is biologically coherent. Most C6 ulcers carry bacterial bioburden; the transition to critical colonisation and biofilm formation sustains a proteolytic, pro-inflammatory wound bed, in which biofilm-resident organisms drive cytokine and MMP release that degrades matrix and inhibits re-epithelialisation, while the biofilm matrix shields bacteria from host clearance and antimicrobial penetration [17,22,24]. Bacterial bioburden has been associated with delayed healing and greater symptom burden in venous ulceration [29,45], and *P. aeruginosa*-colonised wounds—with *P. aeruginosa* being the most frequently identified organism among patients with wound infection (18.3%)—tend to be larger and to heal poorly [21]. The convergent finding that microbial eczema carried a similar unadjusted estimate (OR 5.10), and that the two variables were strongly collinear (Cramér’s V = 0.730), suggests that both are capturing a shared underlying construct of clinically significant wound bioburden rather than two independent processes.

Two alternative explanations must be considered. First, the direction of the association cannot be established from these data: larger, longer-standing or otherwise more refractory ulcers may simply be more prone to wound infection, so that wound infection may be a consequence of a poor trajectory rather than a driver of it. The observation that the duration of the current ulcer was numerically much longer in the unfavourable group (24.69 versus 5.92 months), albeit without reaching significance (*p* = 0.112), is consistent with this possibility. Second, wound infection was identified retrospectively from the clinical diagnosis documented by the treating physician, with culture providing supportive microbiological information where sampling was performed. Because prospectively standardised diagnostic criteria were not available, some degree of misclassification between colonisation and true clinical infection cannot be excluded.

With those caveats, the finding supports an infection- and wound-bed-focused management pathway. Debridement to remove devitalised tissue and disrupt biofilm remains a cornerstone of wound-bed preparation [46,47], complemented by attention to peri-ulcer skin [48], which is particularly relevant in a cohort where eczema was present in 81.6%. Systemic antibiotics should be reserved for clinical infection and guided by culture and antibiogram, since routine use confers little healing benefit and selects resistance [49]; agents with MMP-modulating properties such as doxycycline have been explored as adjuncts [50]. Compression remains the foundation of venous-ulcer therapy and recurrence prevention [51,52], with adjuvant options—foam sclerotherapy with or without ligation [53], negative-pressure wound therapy [54,55], exercise and nutritional optimisation [35,56], prolotherapy [57], and, for correctable reflux, endovascular or surgical intervention [58]—selected according to current guidelines [4,7,59].

The resistance profile in this cohort was reassuring rather than alarming: among isolates obtained from patients with wound infection, 80.0% were multi-sensitive and 13.3% were multidrug-resistant, proportions comparable to those reported in other chronic and ischaemic leg ulcer series [27,28,60]. The higher multidrug-resistant proportion among the eight patients with cellulitis (37.5%) is based on too few patients to be interpreted.

### 4.2. Multiple Ulcerations

Multiple ulceration was independently associated with unfavourable evolution (adjusted OR 5.01), whereas neither circumferential morphology nor maximum ulcer diameter showed any association. This dissociation is plausible: a greater number of discrete lesions implies a larger total area of compromised skin, a more extensive field of microangiopathic and lipodermatosclerotic change, and a longer aggregate epithelialisation front, none of which is captured by the diameter of the single largest lesion. It is also consistent with the recorded reasons for deterioration in this cohort, where an increase in the number of ulcers was the commonest documented explanation for worsening (3/7 patients). Multiple ulceration is a simple bedside observation and, if confirmed in larger series, could contribute usefully to admission-level risk stratification. The wide confidence interval (1.12–22.46) means the magnitude of this association is poorly estimated and only its direction should be regarded as established.

### 4.3. Robustness to Incomplete Outcome Ascertainment

Because outcome was undocumented in a third of the cohort, the credibility of every association depends on how the undocumented patients would have fared. The tipping-point analysis converts this from a rhetorical limitation into a quantitative one, and the answer differs sharply between variables.

For wound infection the bias required to overturn the finding would represent a marked and arguably clinically unlikely departure from the observed data. Nullification demands that undocumented patients with wound infection fared dramatically better than their documented counterparts—an unfavourable rate of 11.7% against an observed 73.7%—despite carrying the same risk factor. If anything, the plausible direction of bias runs the other way: patients who stop attending are more likely to be those doing poorly, which would strengthen rather than weaken the association. The finding therefore does not rest on a missing-at-random assumption.

For multiple ulceration the required departure is larger than would be expected by chance but not inconceivable, and the finding should be regarded as probable rather than secure. For urban residence the required departure is modest—a fall of 23 percentage points—and the association cannot be distinguished from an artefact of differential ascertainment on these data. We therefore treat the infection finding as the study’s principal result, the morphology finding as supported, and the residence finding as provisional. This ordering is reflected in the title and conclusions.

### 4.4. Urban Residence: A Marker, Not a Mechanism

Urban residence carried the highest adjusted odds ratio in the four-predictor model and the second highest in the final model (OR 5.81), a finding for which there is no plausible direct biological mechanism. It should be interpreted as a marker of the pathway by which patients reach and remain in specialist care rather than as a risk factor in any causal sense, and several non-biological explanations deserve consideration.

The most important, which Section 4.3 shows cannot be excluded, is differential outcome ascertainment: if rural patients who do not return are lost to documentation while urban patients who fail to improve continue to attend and accumulate recorded stationary or worsened assessments, the association would arise from the data-capture process alone. Infrequent attendance was itself recorded as the reason for deterioration in 2 of the 7 patients who worsened. A second possibility is referral selection: an urban tertiary centre may receive urban patients later in their course, after failure of local outpatient management, while rural patients present directly. A third is that residence proxies occupational and lifestyle exposures such as prolonged standing, differences in physical activity, or differences in access to and adherence to compression therapy [51,52]. The available data cannot distinguish between these, and the finding should not be transferred into clinical risk assessment without validation in a cohort with complete outcome ascertainment.

### 4.5. Why the Neutrophil-to-Lymphocyte Ratio Was Not Associated with Outcome

The NLR was almost identical in the two outcome groups (3.36 ± 1.82 versus 3.24 ± 1.40; *p* = 0.920), a null result that aligns with broader uncertainty about systemic inflammatory indices in venous disease. Circulating cytokine and chemokine networks are demonstrably altered in chronic venous disease [14,36,37], yet the decisive inflammatory activity in a chronic ulcer is local: wound-fluid cytokines and bioburden, rather than peripheral-blood indices, track healing most closely [29,38,39,40,41].

Several factors plausibly explain the null result here. First, range restriction: the cohort is uniformly C6, so between-stage variance is absent and only within-C6 gradation remains. Second, the NLR reflects systemic rather than local inflammation and is an insensitive proxy for wound-bed activity—a point reinforced by the contrast with CRP, which was associated with outcome on univariate analysis (OR 6.07). Third, the NLR is temporally labile, shifting with intercurrent infection, stress and medication, so a single admission value is noisy. Finally, our other acute-phase markers were recorded only qualitatively, preventing construction of a continuous inflammatory composite [42]. Together these observations suggest that the NLR should not be relied upon to stratify prognosis within established C6 ulceration, although the univariate CRP signal indicates that acute-phase response measured more directly may retain some value and merits quantitative evaluation.

### 4.6. Why Comorbidity Burden Was Not Associated with Outcome

Multimorbidity is repeatedly implicated in venous ulceration, and this cohort confirms its high prevalence: 79.3% had hypertension, 44.8% obesity and 32.2% congestive heart failure. Yet no comorbidity was associated with in-episode outcome, whether analysed as ever-present or as antedating chronic venous disease.

The apparent paradox is resolved by distinguishing between distinct outcomes. Comorbidity—particularly obesity, reduced mobility, lymphoedema and poor nutrition—is linked to ulcer development and to disease progression [32,33,34,35,42], and cardiovascular disease is associated with the presence of venous ulceration [31]. These are determinants of whether and in whom an ulcer arises, and of longer-term recurrence risk, not necessarily of how a given episode evolves once C6 disease is established and the patient is receiving inpatient wound care. Our data are consistent with this separation and do not support the use of a simple comorbidity count as a stand-alone prognostic tool in established C6 disease, while leaving intact its well-established role in primary and secondary prevention. An alternative explanation is statistical: with 25 favourable and 34 unfavourable outcomes, the study had limited power to detect modest comorbidity effects, and these null results should be read as an absence of evidence for large effects rather than as evidence of absence.

### 4.7. Venous Severity Score and Pain

VCSS differed between groups in the expected direction but did not reach significance (14.10 versus 11.07; *p* = 0.090), and VAS pain was essentially identical (5.48 versus 5.55; *p* = 0.977). The VCSS finding is limited by availability, being recorded in only 44 of the 59 patients with documented outcomes, which reduces power substantially; the trend is compatible with a real but modest association that this sample was underpowered to detect. The absence of any relationship with pain is more notable, given that pain has been linked to bacterial load in venous ulcers [45]. It may reflect the qualitative heterogeneity of pain in this population, in which neuropathic, ischaemic and inflammatory components coexist, or the influence of concurrent analgesia, which was not systematically recorded.

### 4.8. Treatment Variables and Confounding by Indication

Five treatment variables were strongly associated with outcome, with systemic antibiotic use showing the largest unadjusted estimate of any variable examined (OR 10.98). These associations almost certainly reflect confounding by indication: systemic antibiotics, iodoform and polarised light were preferentially given to patients whose wounds were infected or failing to progress, so their association with unfavourable evolution reverses the direction of any therapeutic effect. The inverse association observed for instrumental lymphatic drainage (OR 0.17) is subject to the mirror-image bias, since this adjunct is more readily offered to more mobile, less acutely inflamed patients. For these reasons no treatment variable was admitted to the prognostic model, and none of these estimates should be read as evidence of efficacy or harm. Determining the actual effect of these interventions would require randomisation or, at minimum, a design with formal adjustment for treatment indication.

### 4.9. Comparison with the Wider Literature and Clinical Implications

Taken together, these findings place wound-level factors, rather than host-level or haematological ones, at the centre of short-term prognosis in hospitalised C6 disease. This is consistent with the accumulating evidence that local bioburden and wound-bed status govern healing trajectory [21,22,24,29], and with the limited performance of peripheral inflammatory surrogates in this setting [42].

A practical feature of the model is that its three coefficients are almost identical (β = 1.61 to 1.81). A weighted risk equation therefore offers little over a simple count of factors present, each multiplying the odds of an unfavourable course by roughly 5.6. Whether such a count-based score performs adequately cannot be established here—it would require the joint distribution of the three factors and, properly, an independent cohort—but the near-equality of the weights suggests that a three-item tally is worth formal evaluation in future work, since it would be far easier to apply at the bedside than a regression equation.

Practically, the model suggests that two observations available at the bedside within minutes—whether the ulcer is superinfected and whether there is more than one ulcer—carry more prognostic information than the patient’s comorbidity profile or blood counts. If validated, this would support directing intensified wound-bed management, infection surveillance and biofilm-oriented care to patients with these features, and would argue against using systemic inflammatory indices for prognostic triage. The psychosocial burden of C6 disease, including depression, anxiety, pain and impaired quality of life, is substantial and itself influences adherence and outcome [61,62,63]; depression and anxiety were documented in 18.4% and 12.6% of this cohort respectively, reinforcing the value of holistic, infection-aware care even though neither was associated with in-episode outcome.

## 5. Strengths and Limitations

The principal strengths of this study are a consecutive, unselected real-world cohort; a broad variable set spanning demographic, clinical, morphological, microbiological, biological, therapeutic and historical domains; a pre-specified and explicitly documented model-building strategy including collinearity screening and an events-per-variable constraint; and a deliberate exclusion of treatment variables from prognostic modelling.

The limitations are substantial and constrain the strength of any inference. The sample is modest (87 patients, of whom 59 contributed to the prognostic analysis), and the design is observational, retrospective, single-centre and cross-sectional with respect to the outcome, precluding causal inference and limiting generalisability. Outcome was undocumented in 32.2% of patients, and this remains the study’s most serious threat to validity. The tipping-point analysis (Section 3.9 and Section 4.3) bounds its consequences for the univariate associations, but not for the adjusted estimates, which would require the joint distribution of the three factors among the undocumented patients. Although 34 unfavourable events permitted three predictors under a conventional events-per-variable rule, the limiting class in a logistic model is the smaller of the two, and 25 favourable outcomes give a ratio of 8.3 per variable; the confidence intervals around the adjusted odds ratios are correspondingly wide and the effect magnitudes should be regarded as imprecise. The model was not validated internally by bootstrapping or cross-validation, nor externally, so the reported AUC of 0.812 is an apparent, optimistically biased estimate of discrimination; the heuristic shrinkage factor of 0.858 gives an indication of the likely magnitude of coefficient over-fitting but is no substitute for resampling-based optimism correction. A further consequence of the sample size is that the study was powered only to detect large effects: for a comparison of the observed size, the minimum odds ratio detectable at 80% power was approximately 4.7 to 5.2 depending on the baseline event rate. The null findings for comorbidity and for the NLR should therefore be read as excluding large associations only, and are entirely compatible with modest ones.

Acute-phase reactants and metabolic parameters were recorded only as qualitative categories, precluding continuous-biomarker and threshold analyses, and the NLR was the only numeric inflammatory variable. Age was recorded only as a decade band. Standardised ulcer-area measurements were unavailable, so size could be analysed only as a three-level category, and no quantitative measure of bacterial load was obtained; culture data cannot reliably distinguish contamination, colonisation and true infection. Furthermore, because wound infection was retrospectively identified from the treating clinician’s documented diagnosis rather than through prospectively standardised diagnostic criteria, some degree of diagnostic misclassification cannot be excluded. Microbiology fields were hand-entered. We lacked standardised data on compression-therapy adherence [51,52], nutritional status [35] and concomitant lymphoedema [34], each a recognised determinant of healing and each a potential unmeasured confounder. Outcome was assessed at the level of the episode, without longitudinal healing follow-up beyond the eight patients who healed. Finally, the univariate analysis examined a large number of variables without adjustment for multiplicity, so some individual associations may be chance findings; the exploratory stratified analyses are especially vulnerable to this and are reported as hypothesis-generating only.

The study is therefore hypothesis-generating rather than confirmatory. Prospective multicentre studies using standardised ulcer measurement, quantitative inflammatory biomarkers, systematic microbiological sampling, complete outcome ascertainment and formal internal and external model validation are required before these findings can inform practice.

## 6. Conclusions

Among 87 patients admitted with CEAP C6 venous leg ulceration, unfavourable short-term evolution occurred in 57.6% of those with documented outcomes. Wound infection, multiple ulceration and urban residence were each independently associated with an unfavourable course in a three-variable logistic model that explained 40.5% of outcome variance and discriminated acceptably (AUC 0.812). Neither the neutrophil-to-lymphocyte ratio nor any individual comorbidity was associated with outcome, and venous severity as measured by VCSS showed only a non-significant trend.

These are associations observed in a modest, single-centre, retrospective cohort with incomplete outcome ascertainment, and none of them establishes causation. They are not, however, equally secure. Sensitivity analysis shows that the wound infection association would require an implausible pattern of informative missingness to be overturned, whereas the urban-residence association would require only a modest one and is most plausibly explained by referral and documentation patterns rather than by any property of the patients themselves. Within those limits, the findings are consistent with a growing body of evidence that wound-level factors, particularly infection and lesion multiplicity, carry more short-term prognostic information in established C6 disease than systemic inflammatory indices or comorbidity burden. They support prioritising infection surveillance, wound-bed preparation and antimicrobial stewardship in this population, and they require prospective, multicentre confirmation with complete follow-up and formal model validation before any clinical application.

## Figures and Tables

**Figure 1:**
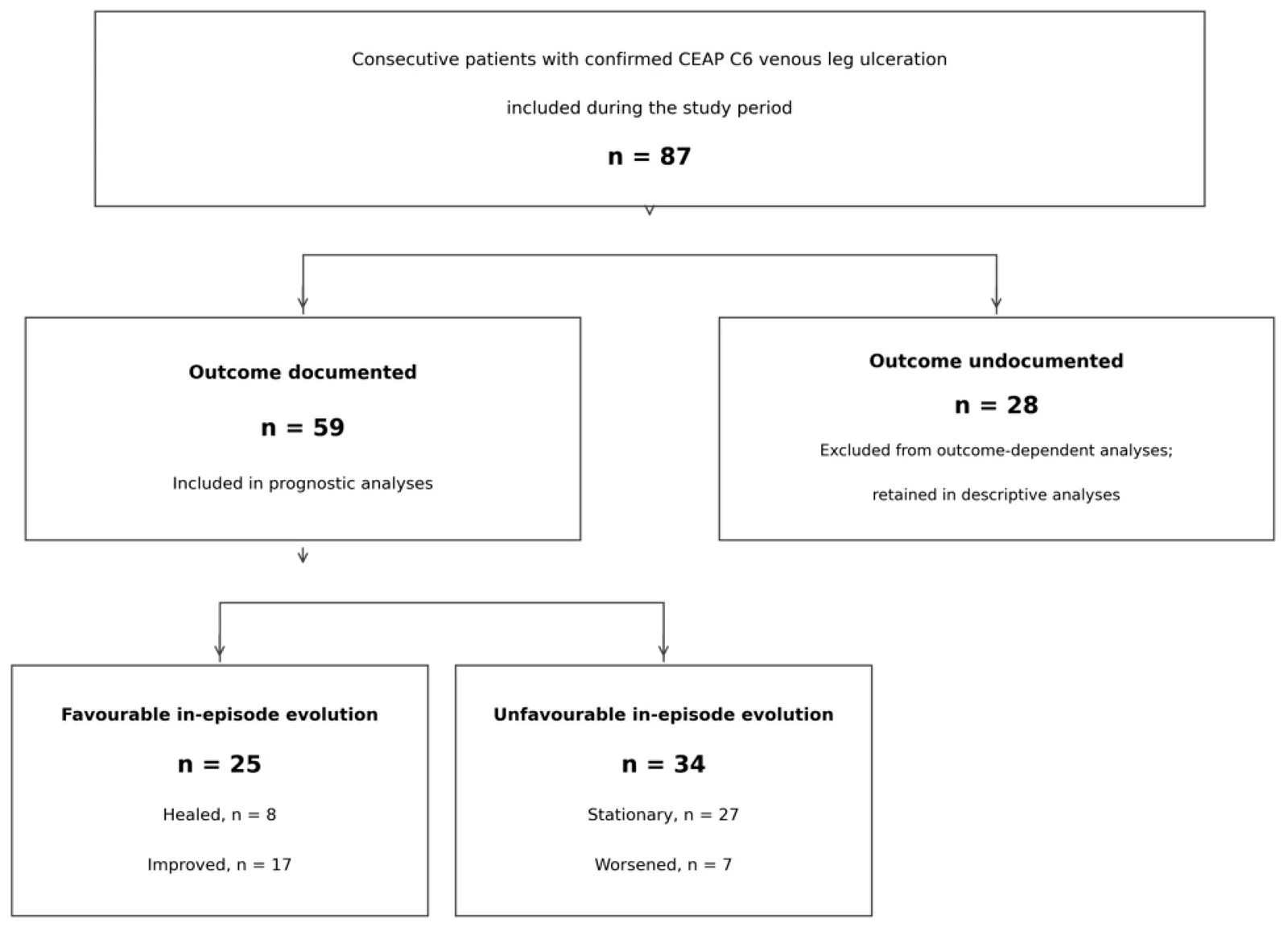
Patient flow.

**Figure 2:**
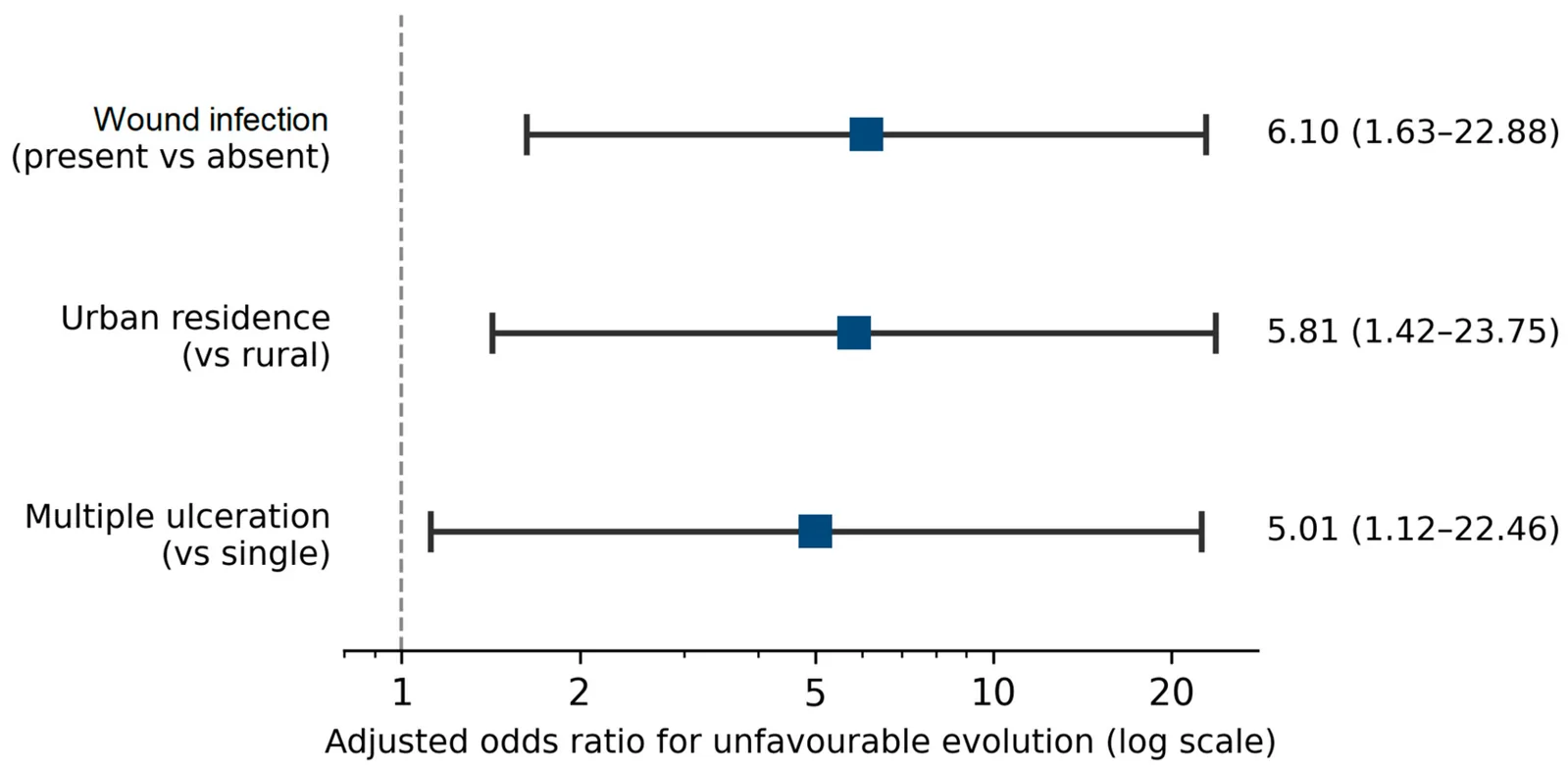
Adjusted odds ratios (95% CI) for unfavourable in-episode evolution in the final three-predictor logistic regression model (*n* = 59). The vertical line indicates the null value (OR = 1); the horizontal axis is logarithmic.

**Figure 3:**
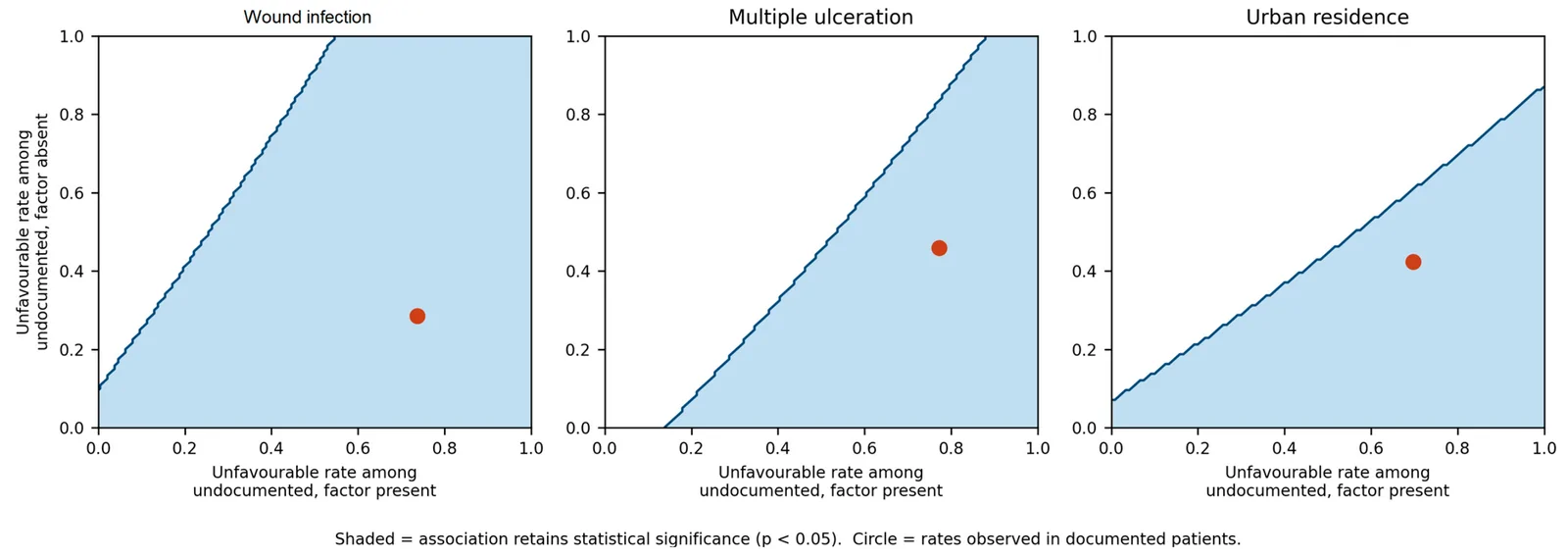
Tipping-point sensitivity analysis for the three model variables. Shaded regions indicate combinations of unfavourable-outcome rates among undocumented patients with and without each factor for which the association retains statistical significance (*p* < 0.05). The circle marks the rates observed among patients with documented outcomes.

**Table 1:** Patient and ulcer characteristics (*n* = 87).

Characteristic	Category	*n*	%
Sex	Male	50	57.5
	Female	37	42.5
Residence	Urban	46	52.9
	Rural	41	47.1
Decade of life	Fourth	4	4.6
	Fifth	12	13.8
	Sixth	21	24.1
	Seventh	29	33.3
	Eighth	18	20.7
	Ninth	3	3.4
Study centre first point of contact	Yes	31	35.6
	No	40	46.0
	Not recorded	16	18.4
Admission type	Continuous inpatient	78	89.7
	Day case	9	10.3
Ulcer number	Single	52	59.8
	Multiple	35	40.2
Circumferential ulceration	Yes	10	11.5
	No	77	88.5
Maximum ulcer diameter	<2 cm	42	48.3
	2–6 cm	24	27.6
	>6 cm	21	24.1

**Table 2:** Continuous clinical variables (*n* = 87 unless stated).

Variable	*n*	Mean ± SD	Median (IQR)	Range
Length of stay (days)	87	6.08 ± 1.76	6.00 (5.00–7.00)	2–11
Number of admissions	87	2.87 ± 2.66	2.00 (1.00–4.00)	1–14
Duration of chronic venous disease (years)	33	28.82 ± 12.28	30.00 (20.00–40.00)	2–47
Time from onset to CEAP C6 (years)	31	23.81 ± 10.98	24.00 (19.00–34.00)	1–40
Duration of current ulcer (months)	62	16.36 ± 25.19	5.50 (2.00–12.50)	0.25–120
VCSS	65	13.20 ± 4.48	11.00 (10.00–16.00)	6–26
VAS pain score	72	5.40 ± 1.93	5.00 (4.00–7.00)	2–9
Neutrophil-to-lymphocyte ratio	84	3.30 ± 1.60	2.93 (2.24–3.95)	0.92–9.34

IQR, interquartile range; SD, standard deviation; VAS, visual analogue scale; VCSS, Venous Clinical Severity Score.

**Table 3:** Microbiological findings by clinical diagnosis.

Finding	Wound Infection (*n* = 60)	Microbial Eczema (*n* = 50)	Cellulitis (*n* = 8)
*Pseudomonas* spp.	11 (18.3)	10 (20.0)	3 (37.5)
MSSA	8 (13.3)	6 (12.0)	2 (25.0)
MSSA + *Pseudomonas*	5 (8.3)	3 (6.0)	2 (25.0)
MRSA	4 (6.7)	3 (6.0)	0 (0.0)
*Escherichia coli*	3 (5.0)	3 (6.0)	0 (0.0)
*Acinetobacter* spp.	3 (5.0)	2 (4.0)	1 (12.5)
*S. aureus*, sensitivity unspecified	3 (5.0)	4 (8.0)	0 (0.0)
Other single or mixed isolates	23 (38.3)	17 (34.0)	0 (0.0)
Antibiogram phenotype			
Multi-sensitive	48 (80.0)	40 (80.0)	5 (62.5)
Multidrug-resistant	8 (13.3)	8 (16.0)	3 (37.5)
Not available	4 (6.7)	2 (4.0)	0 (0.0)

Values are *n* (%) of patients within each diagnosis. Percentages may not sum to 100 owing to rounding. MRSA, methicillin-resistant *Staphylococcus aureus*; MSSA, methicillin-sensitive *Staphylococcus aureus*.

**Table 4:** Univariate analysis of factors associated with unfavourable in-episode evolution (*n* = 59).

Variable	Favourable (*n* = 25)	Unfavourable (*n* = 34)	Test Statistic	*p*	OR (95% CI)
Demographic and ulcer characteristics					
Male sex	15/25 (60.0)	18/34 (52.9)	χ^2^ = 0.291	0.589	—
Urban residence	10/25 (40.0)	23/34 (67.6)	χ^2^ = 4.468	0.035	3.14 (1.07–9.19)
Decade of life	—	—	χ^2^ = 5.143	0.399	—
Day-case admission	2/25 (8.0)	5/34 (14.7)	χ^2^ = 0.620	0.687	—
Multiple ulceration	5/25 (20.0)	17/34 (50.0)	χ^2^ = 5.545	0.019	4.00 (1.22–13.16)
Circumferential ulceration	2/25 (8.0)	5/34 (14.7)	χ^2^ = 0.620	0.687	—
Ulcer size category	—	—	χ^2^ = 2.561	0.278	—
Complications					
Eczema	17/25 (68.0)	28/34 (82.4)	χ^2^ = 1.640	0.200	—
Microbial eczema	8/25 (32.0)	24/34 (70.6)	χ^2^ = 8.643	0.003	5.10 (1.67–15.60)
Wound infection	10/25 (40.0)	28/34 (82.4)	χ^2^ = 11.273	<0.001	7.00 (2.13–23.03)
Cellulitis	1/25 (4.0)	6/34 (17.6)	χ^2^ = 2.566	0.221	—
Laboratory findings					
Elevated ESR	11/23 (47.8)	20/33 (60.6)	χ^2^ = 0.896	0.344	—
Elevated CRP	3/20 (15.0)	15/29 (51.7)	χ^2^ = 6.869	0.009	6.07 (1.46–25.30)
Elevated fibrinogen	2/16 (12.5)	8/19 (42.1)	χ^2^ = 3.730	0.071	—
Elevated fasting glucose	4/24 (16.7)	2/30 (6.7)	χ^2^ = 1.350	0.389	—
Extra-ulcer infectious foci					
Positive nasal swab	1/15 (6.7)	8/21 (38.1)	χ^2^ = 4.610	0.051	—
Other infectious foci	5/20 (25.0)	16/23 (69.6)	χ^2^ = 8.503	0.004	6.86 (1.78–26.36)
Comorbidities					
Hypertension	21/25 (84.0)	28/34 (82.4)	χ^2^ = 0.028	1.000	—
Congestive heart failure	6/25 (24.0)	13/31 (41.9)	χ^2^ = 1.986	0.159	—
Diabetes/impaired glucose	9/25 (36.0)	7/33 (21.2)	χ^2^ = 1.557	0.212	—
Obesity	12/25 (48.0)	16/31 (51.6)	χ^2^ = 0.072	0.788	—
Depression	5/17 (29.4)	9/19 (47.4)	χ^2^ = 1.217	0.270	—
Anxiety	3/17 (17.6)	6/19 (31.6)	χ^2^ = 0.929	0.451	—
Smoking	4/15 (26.7)	3/15 (20.0)	χ^2^ = 0.186	1.000	—
Alcohol use	7/15 (46.7)	2/14 (14.3)	χ^2^ = 3.548	0.109	—
Continuous variables, mean ± SD					
Number of admissions	2.56 ± 1.47	4.50 ± 3.36	U = 258.0	0.009	—
Length of stay (days)	5.72 ± 2.01	6.18 ± 1.36	U = 355.0	0.272	—
Duration of current ulcer (months)	5.92 ± 6.57	24.69 ± 33.64	U = 140.5	0.112	—
VCSS	11.07 ± 2.96	14.10 ± 5.27	U = 149.5	0.090	—
VAS pain score	5.55 ± 2.11	5.48 ± 2.13	U = 308.5	0.977	—
Neutrophil-to-lymphocyte ratio	3.24 ± 1.40	3.36 ± 1.82	U = 373.5	0.920	—
Treatment variables (not eligible for modelling)					
Systemic antibiotics	4/25 (16.0)	23/34 (67.6)	χ^2^ = 15.483	<0.001	10.98 (3.03–39.81)
Iodoform	1/25 (4.0)	11/34 (32.4)	χ^2^ = 7.148	0.009	11.48 (1.37–96.16)
Polarised light	3/25 (12.0)	13/34 (38.2)	χ^2^ = 5.017	0.025	4.54 (1.13–18.24)
Antihistamines	10/25 (40.0)	24/34 (70.6)	χ^2^ = 5.520	0.019	3.60 (1.21–10.69)
Instrumental lymphatic drainage	11/25 (44.0)	4/34 (11.8)	χ^2^ = 7.896	0.005	0.17 (0.05–0.63)

Categorical variables are shown as *n*/*N* (%), where *N* is the number of patients in whom the variable was recorded; continuous variables are mean ± SD. Categorical comparisons used the Pearson chi-squared test; where any expected cell count was below five the reported *p* value derives from Fisher’s exact test, and the Pearson χ^2^ statistic is shown alongside for reference only. Continuous comparisons used the Mann–Whitney U test. Odds ratios are reported for dichotomous variables reaching *p* < 0.05 and refer to the odds of unfavourable evolution. Variables with *p* ≥ 0.10 and no clinical relevance to the model are omitted from this table and are summarised in the text. CI, confidence interval; CRP, C-reactive protein; ESR, erythrocyte sedimentation rate; OR, odds ratio; SD, standard deviation; VAS, visual analogue scale; VCSS, Venous Clinical Severity Score.

**Table 5:** Binary logistic regression models for unfavourable in-episode evolution (*n* = 59).

Predictor	Four-Predictor Model OR (95% CI)	*p*	Final Three-Predictor Model OR (95% CI)	*p*
Urban vs. rural residence	4.76 (1.14–19.88)	0.032	5.81 (1.42–23.75)	0.014
Multiple vs. single ulceration	5.09 (1.12–23.05)	0.035	5.01 (1.12–22.46)	0.035
Wound infection present vs. absent	4.26 (1.05–17.27)	0.042	6.10 (1.63–22.88)	0.007
Number of admissions (per admission)	1.23 (0.90–1.69)	0.196	Not retained	—
Model fit				
Omnibus χ^2^ (df)	23.278 (4)	<0.001	21.176 (3)	<0.001
Hosmer–Lemeshow χ^2^ (df)	5.825 (7)	0.560	4.077 (5)	0.538
Nagelkerke R^2^	0.438	—	0.405	—
Overall classification accuracy	72.9%	—	72.9%	—
Accuracy, favourable/unfavourable	68.0%/76.5%	—	72.0%/73.5%	—

Binary logistic regression, enter method; the null model correctly classified 57.6% of cases. CI, confidence interval; df, degrees of freedom; OR, odds ratio.

**Table 6:** Tipping-point sensitivity analysis for undocumented outcomes (28 patients).

Variable	Undocumented Patients with/Without Factor	Observed Unfavourable Rate, Factor Present	Nullified When Rate Falls to	Observed Unfavourable Rate, Factor Absent	Nullified When Rate Rises to	Scenario Space Retained
Wound infection	22/6	73.7%	11.7%	28.6%	Not nullified at 100%	74.4%
Multiple ulceration	13/15	77.3%	50.3%	45.9%	83.9%	47.4%
Urban residence	13/15	69.7%	46.7%	42.3%	60.8%	45.5%

## Data Availability

The de-identified dataset analysed in this study is available from the corresponding author upon reasonable request.
